# Synthesis of a New Glycoconjugate with Di-*ᴅ*-Psicose Anhydride Structure

**DOI:** 10.3390/ijms232112827

**Published:** 2022-10-24

**Authors:** Young Sung Jung, Hyoung-Geun Kim, Min-Cheol Lim, Ji-Su Park, Soonok Sa, Miyoung Yoo

**Affiliations:** 1 Korea Food Research Institute, Wanju 55365, Korea; 2 Graduate School of Biotechnology and Department of Oriental Medicinal Biotechnology, Kyung Hee University, Yongin 17104, Korea; 3 Food Biotech R&D Center, Samyang Corp., Seongnam 13488, Korea

**Keywords:** anhydrous acidification, *ᴅ*-allulose, caramelization, metal chelation, spiro-tricyclic disaccharide

## Abstract

Demand for healthy diets has led researchers to explore new saccharide as sucrose alternatives. *ᴅ*-Psicose, the C-3 epimer of *ᴅ*-fructose, has a similar sweetness intensity to sucrose but contributes fewer calories. This study proposes a disaccharide with a stable structure derived from *ᴅ*-psicose. The compound with a spiro-tricyclic core was generated at 32% conversion via caramelization of *ᴅ*-psicose under acidic anhydrous conditions. The compound was identified by high-resolution mass spectrometry and multi-dimensional nuclear magnetic resonance (NMR). The molecular formula was established as C_12_H_20_O_10_ from the molecular weight of *m*/*z* 324.1055. Twelve signals were observed by the ^13^C NMR spectrum. This compound, denoted di-*ᴅ*-psicose anhydride (DPA), exhibited a lower water solubility (40 g/L) and higher thermal stability (peak temperature = 194.7 °C) than that of *ᴅ*-psicose (peak temperature = 126.5 °C). The quantitatively evaluated metal ion scavenging ability of DPA was the best in magnesium (average 98.6 ± 1.1%). This synthesis methodology can provide disaccharides with high stability-reducing heavy metals.

## 1. Introduction

Sugars satisfy an innate human preference for sweet tastes [1,2], and also provide an energy source in the human diet [2,3]. However, excess sugar intake can accumulate body fat and damaging micronutrients. Over the past two decades, the demand of consumers for reduced-sugar products to achieve healthier diets has increased. This demand has led researchers to explore new natural and synthetic disaccharides as alternatives to sucrose and fructose [2,3].

*ᴅ*-Psicose is the C-3 epimer of *ᴅ*-fructose, which is rarely found in nature [4]. According to the International Union of Pure and Applied Chemistry, *ᴅ*-psicose is systematically named *ᴅ*-ribo-2-hexulose. The term “psicose” is derived from the antibiotic psicofuranine [5]. *ᴅ*-Psicose has a sweetness intensity (70%) similar to sucrose, but contributes fewer calories (0.4 kcal/g) owing to its slower and incomplete absorption in the intestine [2,6]. In addition, *ᴅ*-psicose can form fructose-derived difructose anhydride (DFA) structures [7,8].

*ᴅ*-Psicose can dehydrate two water molecules to form a spiro-tricyclic-disaccharide [7]. The finding of DFA derived from fructose (an isomer of *ᴅ*-psicose) supports this hypothesis [8,9]. This cyclic fructo-disaccharide has the characteristics of a low-calorie disaccharide that is highly stable in strong acids at high temperatures [10]. DFA is obtained chemically by the thermal treatment of inulin or *ᴅ*-fructose in the presence of an acid catalyst [11]. To date, thirteen DFA isomers with five structurally different tricyclic cores have been identified from fructose caramel [12]. However, *ᴅ*-psicose-derived pseudodisaccharides based on precise identification of structure have not yet been reported.

In this study, *ᴅ*-psicose was caramelized to prepare a disaccharide with a dianhydride structure. The structure of the isolated compound was identified by high-resolution mass spectrometry (HRMS) and multi-dimensional nuclear magnetic resonance (NMR) spectroscopy. The compound, denoted di-*ᴅ*-psicose anhydride (DPA), was found to differ from *ᴅ*-psicose through differential scanning calorimetry (DSC), and the metal ion reduction effect was quantitatively evaluated in molar units. This study presents an academic approach for the development of a pseudodisaccharide derived from *ᴅ*-psicose with high structural stability.

## 2. Results

### 2.1. Anhydrous Acidification

**Figure 1:**
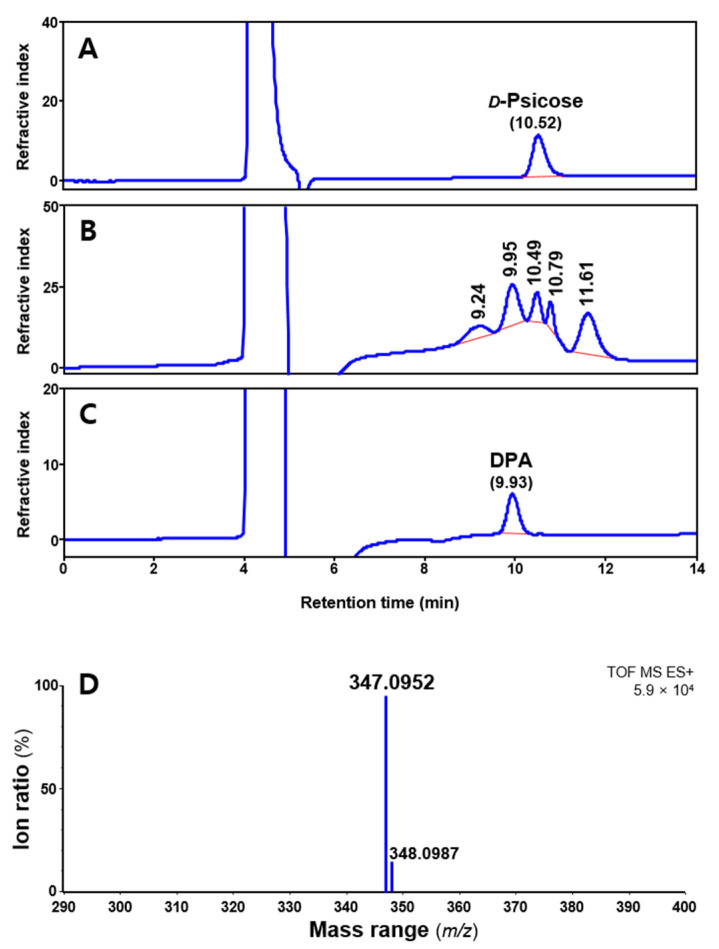
High-performance liquid chromatography chromatograms of *ᴅ*-psicose standard (**A**), acidify reactant (**B**), isolated single compound (**C**), and high-resolution mass spectrometry spectrum of di-*ᴅ*-psicose anhydride (DPA) (**D**).

Anhydrous acidification was performed using undiluted hydrochloric acid (over 12 M). In preliminary experiments, hydrochloric acid with a concentration below 6 M did not react at −20 or 4 °C. The generated peak and remaining *ᴅ*-psicose after the anhydrous acidification were quantified by high-performance liquid chromatography (HPLC) equipped with a refractive index detector (Figure 1). After reaction, four peaks at 9.2, 9.9, 10.8, and 11.6 min were generated for all samples, except *ᴅ*-psicose (10.5 min). Among them, the retention time of the largest peak was 9.9 min, which was consistent with the identified compound.

Table 1 presents the effects of reaction time and temperature on DPA and *ᴅ*-psicose concentrations. All reactants at 40 °C were dehydrated and insoluble in dimethyl sulfoxide (DMSO). Similarly, *ᴅ*-psicose reacted at 25 °C for more than 4 days and was not dissolved (Figure S1); thus, the corresponding results are indicated as “N.A” in Table 1. The ratio of the produced DPA to the reduced *ᴅ*-psicose at −20 °C and 4 °C is presented in Figure S2. The temperature conditions significantly (*p* < 0.05) increased the DPA production. In particular, the DPA produced at 4 °C (average 679.3 ± 134.7 mM) was 26.9% more than that at −20 °C (average 496.8 ± 134.5 mM). 

At 4 °C, the DPA production increased proportionally with the reaction time. The highest DPA content with a conversion rate corresponding to 32% of the input *ᴅ*-psicose was generated at 8 days. In the reactants, the remaining *ᴅ*-psicose decreased significantly (*p* < 0.05) with the increase in temperature and reaction time, which is consistent with the total peaks (orange and blue boxes) in Figure S3.

### 2.2. Structure Identification

**Figure 2:**
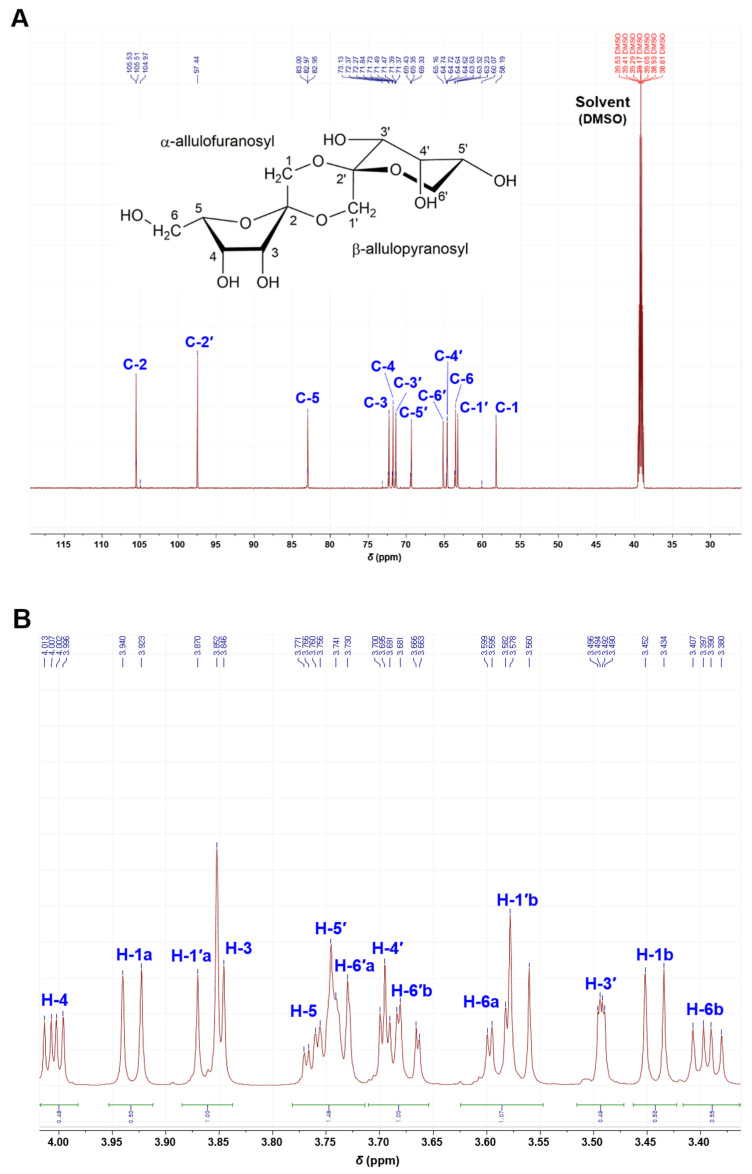
^13^C NMR spectrum (**A**) and ^1^H NMR spectrum (**B**) of di-*ᴅ*-psicose anhydride.

The isolated compound was identified using HRMS and NMR. The molecular formula was established as C_12_H_20_O_10_ from the molecular ion peak [M + Na]^+^ *m*/*z* 347.0952 (calcd. *m*/*z* 347.0954 for C_12_H_20_O_10_Na, error = 0.6 ppm) (Figure 1D). The unknown peak at 11.6 min could not be measured. The ^13^C NMR spectrum (Figure 2A), supported by distortionless enhancement by polarization transfer (DEPT) spectrum (Figure S4), indicated twelve carbon signals confirming the compound to be composed of two hemiketals [δ_C_ 105.86 (C-2), 97.80 (C-2′)], six oxygenated methines [δ_C_ 83.30 (C-5), 72.62 (C-3), 72.08 (C-4), 71.72 (C-3′), 69.68 (C-5′), 64.97 (C-4′)], and four oxygenated-methylene [δ_C_ 65.51 (C-6′), 63.87 (C-6), 63.58 (C-1′), 58.54 (C-1)]. The chemical shifts of carbon signals at C-1 to C-6 and C-1′ to C-6′ are shown in the ^13^C NMR spectrum. In Figure 2B, the ^1^H NMR signals were observed, except for C-2 and C-2′, which do not allow C-H bonding. 

**Figure 3:**
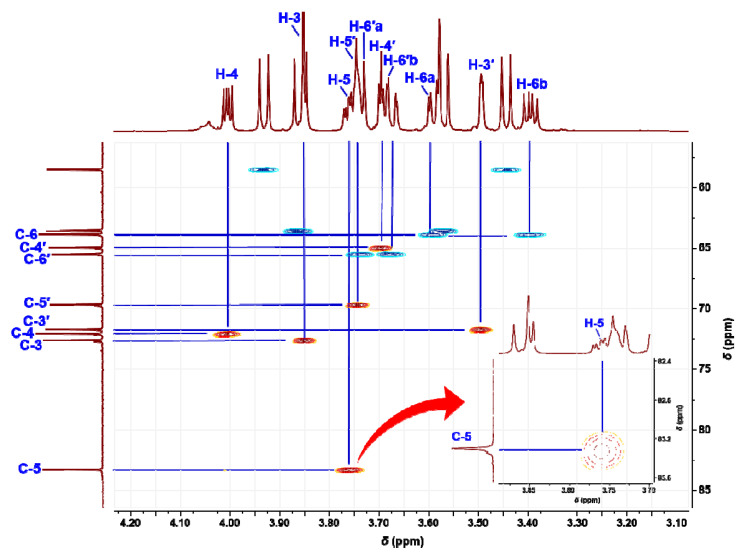
Heteronuclear single quantum coherence spectrum of di-*ᴅ*-psicose anhydride.

**Figure 4:**
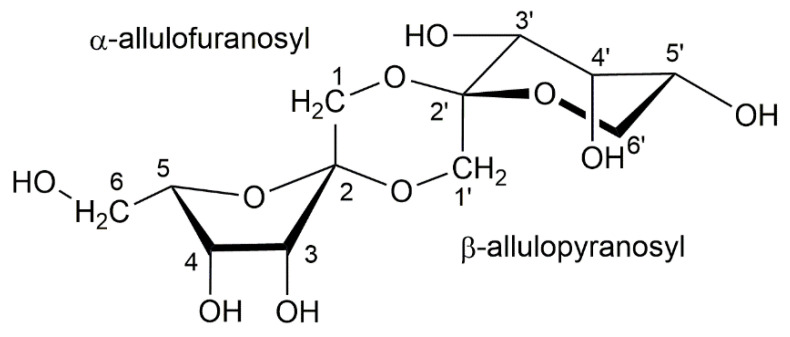
Molecular structure of di-*ᴅ*-psicose anhydride.

In the heteronuclear single quantum coherence (HSQC) spectrum (Figure 3), δ_H_ 3.75 (1H, ddd, *J* = 7.7, 7.0, 2.8 Hz) of the cross peak with a C-5 signal was observed as an H-5 signal of *α*-*ᴅ*-psicofuranose (starting compound). The two-dimensional NMR spectra are shown in Figures S5 and S6. In the HSQC spectrum of the isolated compounds and the assignments of the ^1^H NMR signals were unambiguously confirmed. In particular, the H-6′ signal of ketohexose showed a unique coupling patterns, such as doublets of doublets and broad doublets. Several cross-peaks in the ^1^H−^1^H correlation spectroscopy spectrum confirmed a key correlation among the proton signals. The heteronuclear multiple bond correlation (HMBC) spectrum showed ^3^*J* correlations between H-1/C-3, C-2′ and H-1′/C-3′, C-2, and showed ^2^*J* correlations between H-1/C-2 and H-1′/C-2′, respectively. The structure of the isolated compound based on the NMR spectra and HRMS spectrum is shown in Figure 4.

### 2.3. Water Solubility and Endothermic Heat Flow

DPA was obtained as a white odorless powder. Its water solubility was determined by HPLC at room temperature (25 °C). After centrifugation, the initial 3.55 mM DPA solution was quantified to be 2.23 mM in the supernatant, incubating approximately 40 g DPA dissolved in 1.0 L water. 

**Figure 5:**
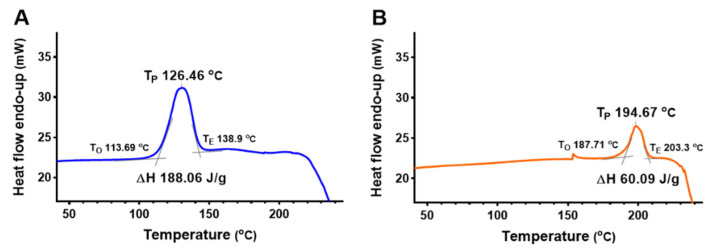
Differential scanning calorimetry profiles of *ᴅ*-psicose (**A**) and di-*ᴅ*-psicose anhydride (**B**).

Figure 5 shows the change in the endothermic heat flow of DPA and *ᴅ*-psicose at room temperature. The peak temperature (T_P_), onset temperature (T_O_), and endset temperature (T_E_) were determined. The T_O_ of DPA was approximately 74 °C higher than that of *ᴅ*-psicose. Similarly, DPA had a higher T_P_ at 68 °C. During melting, the ΔH of DPA was approximately 32% of the value of *ᴅ*-psicose.

### 2.4. Metal Ion Reduction

**Figure 6:**
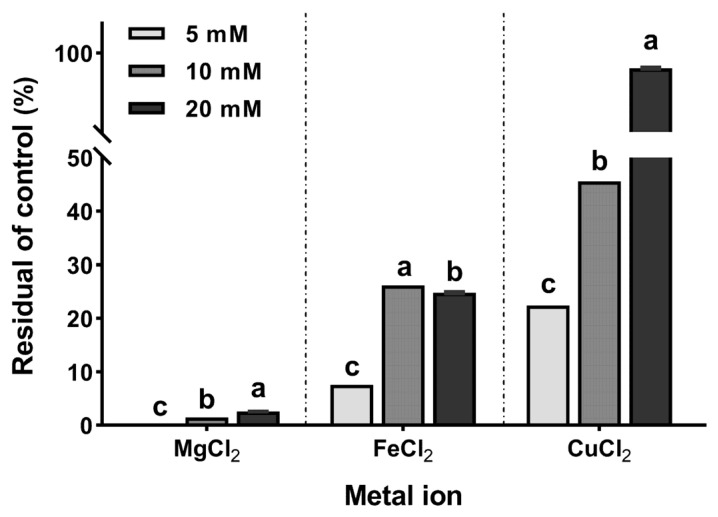
Reduction rate of the metal ion concentrations with di-*ᴅ*-psicose anhydride (10 mM). Different lowercase letters on bars in the same metal ion indicate significant differences by Tukey–Kramer honestly significant difference test (*p* < 0.05).

Figure 6 shows the amount of metal ions reduced after incubation with 10 mM DPA. One-way analysis of variance (*p* < 0.01) was performed based on the concentration by classifying the metal ions. The magnesium ions exhibited the largest reduction (average reduction 98.6 ± 1.1%). The iron ions exhibited the largest reduction at the concentration of 5 mM (92.4 ± 0.3%); however, there was no concentration-dependence observed. In particular, iron ions at 20 mM (78.3 ± 0.5%) had larger reductions than that at 10 mM (75.2 ± 0.5%). Copper ions had the lowest reduction obtained at 20 mM (7.7 ± 0.5%). In summary, the metal ions significantly reduced (*p* < 0.001) in the order of Mg > Fe > Cu at 10 mM, which was similar to the molar ratio of incubated DPA.

## 3. Discussion

DPA is a spiro-tricyclic psicodisaccharide consisting of two *ᴅ*-psicose units linked together with two anhydride bonds (Figure 4). This structure is well known in DFAs derived from fructose monomer, which is an isomer of *ᴅ*-psicose [9,10,12]. Thus, this confirms that DPA was derived from *ᴅ*-psicose. DFAs are generated through thermal and/or acidic treatments, and are mostly made from substances that exist in nature, such as inulin and levan [7,12,13,14]. However, the polymerization of *ᴅ*-psicose remains a huge challenge. This study explored the direct generation of anhydride bonds to *ᴅ*-psicose, which was expected to reduce the processing step, increase the reaction efficiency, and increase product yield. Consequently, the methodology in this study allows for a DPA conversion of 32% from *ᴅ*-psicose.

Anhydrous acidification is used to caramelize a ketohexose by generating a cation [9,13]. The cation produced by the hydrochloric acid can react with nucleophiles, such as hydroxyl groups attached to sugar [13]. According to the accepted mechanism for dianhydride formation, two *ᴅ*-psicoses comprise a psicosyl oxocarbenium cation that undergoes in situ glycosylation to form a ketodisaccharide. Subsequently, the transient ketodisaccharide is formed and undergoes intramolecular spiroketalization. Further intramolecular spiroketalization closes the central 1,4-dioxane ring. This process is reversible and dependent on the products in proportion with time, temperature, and initial concentration [7,15].

The effect of the reaction temperature and time on DPA production is shown in Table 1 and Figure S1. Temperatures higher than 25 °C rapidly dehydrated the reactants, which hindered the neutralization and analysis of DPA. In particular, the reactants were solidified at 40 °C within one day. To address this issue, hydrochloric acid was diluted to 6.0 M using water. However, there was still no considerable progress on the caramelization (data not shown). In addition to DPA, *ᴅ*-psicose was converted to other compounds with peaks at 9.24, 10.79, and 11.61 min, which can be ascribed to the aforementioned reversible spiroketalization. In the case of DFA, 15 isomers have been characterized; of which, 13 have been identified from fructose caramel [8]. Therefore, peaks generated at the other retention times are inferred as isomers of DPA.

For the DPA generation, temperature was noted to have a more pronounced effect than reaction time. In particular, the DPA obtained at 4 °C and one day had no significant difference (*p* > 0.05) to that obtained at 25 °C and 6 days. This indicates that increasing the temperature can reduce the reaction time. Therefore, the optimal reaction temperature was noted to be higher than 4 °C. Considering the temperature, reaction time, and dehydration rate, DPA can be obtained under the optimal reaction conditions of 10–20 °C and 48 h.

*ᴅ*-Psicose acidified with hydrochloric acid generates equal molecular weights (*m*/*z* 324.1055 = *m*/*z* 347.0952 − Na^+^) of the peak (9.95 min) as that of DFA (Figure 1). The molecular weight of *m*/*z* 324 comprises two water (*m*/*z* 36) dehydrated from two *ᴅ*-psicoses (*m*/*z* 360), which corresponds well with the NMR spectra. The twelve signals observed in the ^13^C NMR spectrum (Figure 2A) indicate that there are two ketohexoses and two anhydride bonds between them. The presence or absence of signals near 75 or 85 ppm are determinants for the two ketohexoses constituting DPA. The absence of C-2 and C-2′ signals in the DEPT-135 spectrum (Figure S4) confirmed that the two ketohexoses of DPA were *ᴅ*-psicose, because both of the two signals were bonded by an element other than hydrogen.

*ᴅ*-Psicose (starting substance) can exist in four formations, namely *α*-*ᴅ*-psicofuranose, *β*-*ᴅ*-psicofuranose, *α*-*ᴅ*-psicopyranose, and *β*-*ᴅ*-psicopyranose by mutarotation in solution (in a ratio of 38:13:25:24) [16]. Among them, only *α*-*ᴅ*-psicofuranose was observed at low magnetic field, approximately 85 ppm in ^13^C NMR, and there was no signal at 75 ppm. In the HSQC spectrum (Figure 3), the first ketohexose was confirmed as *α*-*ᴅ*-psicofuranose because the δ_H_ 83.30 (C-5) signal showed a cross-peak with δ_H_ 3.75 (1H, ddd, *J* = 7.7, 2.8, 7.0 Hz), the commonly reported signal of H-5 from *α*-*ᴅ*-psicofuranose. In addition, the coupling pattern (dd or broad-d) of the H-6′ signal in the ^1^H NMR spectrum (Figure 2B) indicated that the second ketohexose was *β*-*ᴅ*-psicopyranose. In the HMBC spectrum (Figure S5) confirmed that two ketohexoses, *α*-*ᴅ*-psicofuranose and *β*-*ᴅ*-psicopyranose, were combined at A1-*O*-B2 and B1-*O*-A2 and formed a hexagon. From these NMR spectra and the molecular weight (*m*/*z* 324.1055), the structure of the isolated compound (peak 9.95 min) was identified as a novel compound, denoted di-*ᴅ*-psicose anhydride (Figure 1). As mentioned earlier, the unidentified peaks (10.79 min and 11.61 min) were expected to be isomers of DPA (e.g., DFA I–IV) or compounds to which three *ᴅ*-psicose were bound [8,13,17].

The water solubility of DPA (about 4 g/100 mL) is lower than that of *ᴅ*-psicose (291 g/100 mL) [18]. Similarly, sucrose, which is an ester-bonded disaccharide of fructose and glucose, is confirmed to be a disaccharide with lower water solubility than its monosaccharides [19,20]. This result is also supported by DFA derived from fructose. In particular, the water solubility of DFA III is 60% (*w*/*w*), which is 90–95% of the water solubility of sugar [12,21]. As DPA is produced by the dehydration condensation of the hydroxyl groups of *ᴅ*-psicose, its polarity is expected to decrease during this process.

DSC analysis is intrinsically dynamic and it is impossible to sense equilibrium [22]. In this study, *ᴅ*-psicose and DPA were measured through DSC for compatibility with other studies. Figure 5 shows the change in the enthalpy of DPA at a temperature higher than that of *ᴅ*-psicose. According to the study on the heat capacity of rare sugars, physical stability increases when two ketohexoses are combined in an anhydride structure [10]. In addition, the five hydroxyl groups of ketohexose cause different intermolecular hydrogen bonding. The intramolecular hydrogen bond stabilizes the ring conformation of *ᴅ*-psicose, even in its crystalline state.

The intramolecular hydrogen bonding in *ᴅ*-psicose can stabilize the ring conformation of ketohexoses. *ᴅ*-Psicose has more intramolecular hydrogen bonds than crystalline fructose and tagatose [10]. The thermal stability of DPA (T_P_ at 194 °C) suggests its potential as a food additive. *ᴅ*-Psicose, a monomer of DPA, increases the moisture content and reduces the tendency to crystallize in gelatin-based soft candy. The high-water association of *ᴅ*-psicose decreases the glass transition temperature value owing to the plasticizer effect [23].

The DPA structure has an additional six-membered ring between the two ketoses. The 1,4-dioxane in the center of this spiro-tricyclic psicodisaccharide has a boat conformation, which participates in the complex stabilization with a metal cation. Together with the two oxygen atoms of 1,4-dioxane, the oxygen atoms of the hydroxyl groups at the C-3 and C-3′ signals simultaneously promote the bonding of the metal cation [7,24]. This disaccharide with a dianhydride structure indicates the potential of DPA to be involved in the absorption of metal cations into the human body [8,12].

The reduction rate of DPA for each metal ion can be related to the electrochemical properties of the metal ions (Figure 6). The standard electrode potential reflects the reaction of the metal ions in an aqueous solution. In addition, it represents the ability of the metal ions to react more easily to form compounds. The standard electrode potential of magnesium, iron, and copper are −2.37 V, −0.45 V, and +0.15 V, respectively [25,26]. This standard electrode potential trend is consistent with the reduction rate of DPA. Therefore, DPA can be specialized in reducing metal ions with low standard electrode potential. Studies on DFA, which has the same structure as DPA and promotes the absorption of minerals including calcium ion (−2.87 V) in the small intestine, have reported that it appears to have a high correlation with the metal ion binding properties of DPA [8,27].

## 4. Materials and Methods

### 4.1. Chemicals and Reagents

Analytical grade *ᴅ*-psicose (≥95%), hydrochloric acid (37%), sodium hydroxide, DMSO, iron(II) chloride (≥99%), magnesium chloride (≥99%), copper(II) chloride (≥99%), sodium phosphate monobasic, and sodium phosphate dibasic were purchased from Sigma Aldrich (St. Louis, MO, USA). Industrial grade *ᴅ*-psicose (≥95%) was obtained from Hengshui Haoye Chemical (Hebei, China). HPLC grade water, methanol, and acetonitrile were purchased from Thermo Fisher Scientific (Waltham, MA, USA).

### 4.2. Anhydrous Acidification

Anhydrous acidified *ᴅ*-psicose was inspired by previous studies [9,13]. To synthesize psicofuranosyl cations, 10 g *ᴅ*-psicose was dissolved in 10 mL cold (4 °C) hydrochloric acid by ultra-sonication for 5 min. The reaction was stored at −20, 4, 25, and 40 °C for 1, 2, 4, 6, and 8 days, respectively. The reaction was stopped by adding equal volumes of DMSO, vortexed for 1 min, and sonicated for 5 min. For the HPLC analysis, the reactants were diluted five-fold with 1 N sodium hydroxide and five-fold with 50% (*v*/*v*) methanol in water (Figure S1).

### 4.3. HPLC Analysis

The samples were analyzed using a reversed-phase HPLC system (Chromaster^®^; Hitachi High-Tech, Tokyo, Japan) equipped with a refractive index detector (5450; Hitachi High-Tech) and a Shodex Asahipak NH_2_P-50 4E C_18_ column (4.6 mm × 250 mm, 4.5 μm; Shodex, Kanagawa, Japan). The mobile phase was 75% (*v*/*v*) acetonitrile in water. The total isocratic elution run time was 15 min. The flow rate and injection volume were 0.6 mL/min and 20 μL, respectively. The column oven temperature was set to 35 °C. Before the analysis, the samples were filtered through a 0.2 μm polytetrafluoroethylene filter (Nanosep^®^; Pall Corp., Port Washington, NY, USA).

### 4.4. Preparation of Single Compound

For the HRMS and NMR analysis, the sample was filtrated using Whatman no.4 filter paper (Whatman International Ltd., Maidstone, UK) under vacuum conditions. The filtered solution was performed using a preparative HPLC system (JAI LC-9104; Japan Analytical Industry, Tokyo Japan), equipped with a refractive index detector (Japan Analytical Industry). Separation was achieved on a YMC-Pack Polyamine II C_18_ column (250 mm × 20 mm, 5.0 μm; Kyoto, Japan). The mobile phase was 78% (*v*/*v*) acetonitrile in water. The isocratic elution was carried out at 30 °C at a run time of 30 min. The flow rate and injection volume were 10 mL/min and 5 mL, respectively. The effluent was collected (4.0 mL each) according to the elution profile using a fraction collector (DC-1500; Eyela, Tokyo, Japan). The collected solvent underwent vacuum evaporation (N-1000; Eyela) at 40 °C in a water bath.

### 4.5. Structure Identification

The molecular weight of a single fraction was analyzed using a time-of-flight HRMS system (SYNAPT G2-HDMS; Waters Corp., Manchester, UK) with an electrospray ionization source operated via MassLynx 4.1 software. The sample was directly infused into the mass spectrometer through the electrospray ionization source. The mass spectrum was acquired in the positive ion mode and recorded in the range of 50–1200 *m*/*z*. The HRMS parameters were optimized as follows: capillary voltage of 2.5 kV, cone voltage of 40 V, source temperature of 120 °C, and desolvation gas flow rate of 500 L/h.

The obtained single compound was dissolved in 100 μL DMSO-*d*_6_. NMR analysis was conducted using a Bruker 600 MHz (Bruker Avance 600; Bruker GmBH, Rheinstetten, Germany). One-dimension NMR analysis was performed for ^1^H (600 MHz), ^13^C (150 MHz), and DEPT. Two-dimension NMR spectra were recorded for the HSQC, correlation spectroscopy, and HMBC. All chemical shifts were given in ppm. The structure was assigned using MestreNova 11.0 (Mestrelab Research SL, Santiago de Compostela, Spain).

### 4.6. Compound Properties

The assessment of the water solubility of the DPA followed the method described in a previous study [28]. First, 6.4 mg DPA with 100 μL deionized water was vortexed for 5 min and incubated in an ultrasonic water bath (Power sonic 420; Hwashin, Seoul, Korea) for 30 min at 25 °C. The DPA solution was centrifuged (5180; Eppendorff, Hamburg, Germany) at 20,376× *g* and 4 °C for 10 min. The supernatant of the DPA solution was filtered through a 0.2 μm polytetrafluoroethylene filter (Nanosep^®^). The amount of DPA was analyzed using HPLC.

The DSC experiments were carried out using a DSC 4000 (Perkin Elmer, Waltham, MA, USA), equipped with a cooling system. DPA and *ᴅ*-psicose were weighed to 4.5 mg, respectively, in a hermetic aluminum pan. The aluminum pans were sealed and placed in a measuring cell. An empty aluminum pan was used as the reference. The endotherm scans of the samples were obtained at a rate of 10 °C/min from 25 to 300 °C. Dry nitrogen was used to purge the sample at 20 mL/min.

### 4.7. Metal Ion Reduction

The metal ions FeCl_2_, MgCl_2_, and CuCl_2_ were dissolved in deionized water at concentrations of 5, 10, and 20 mM, respectively. DPA was prepared in deionized water and diluted with the same solvent at 10 mM. Subsequently, 100 μL of DPA and metal ions was mixed and vortexed for 1 min. The mixed reactants were incubated in a thermomixer (Thermo Fisher Scientific) for 24 h at 37 °C and 400 rpm. The reacted solutions were diluted with a 2% (*v*/*v*) aqueous nitric acid solution before the metal ion analysis. The metal ion analysis was performed using inductively coupled plasma with an optical emission spectroscopy system (AVIO 500; Perkin Elmer). The three metal ions were quantified using the standard curve of each standard.

### 4.8. Data Expression and Statistical Analysis

The experiments were conducted in triplicate. The data were expressed as the mean ± the standard deviation of the values. The graphs were generated and drawn using GraphPad Prism 8.4.3 for Windows (GraphPad, Inc., San Diego, CA, USA). Statistical analysis was performed using JMP^®^ 15 statistical software (SAS Institute Inc., Cary, NC, USA). One-way and two-way analyses of variance followed by the Tukey’s honestly significant difference test (*p* < 0.05) were applied to determine the significance of the differences among the means.

## 5. Conclusions

*ᴅ*-Psicose-derived compound with a spiro-tricyclic-disaccharide structure was obtained via a caramelization reaction. The compound, with a molecular weight of 324.1055, was identified as DPA via NMR analysis. Temperature and reaction time were found to be important factors for DPA production. The most DPA was produced (conversion of 32%) at 10–20 °C and 48 h. DPA has a lower water solubility than *ᴅ*-psicose, but has higher thermal stability. The spiro-tricyclic structure can bind metal ions (magnesium > iron > copper) indicating the potential of DPA for hazardous substance removal or mineral transport. The current study still has a limitation in that an efficient DPA production and purification methodology was not achieved. Nevertheless, this synthesis methodology can provide disaccharides with high stability, reducing heavy metals.

## 6. Patents

Korea Food Research Institute has filed a patent (10-2022-0018883) that describes the synthesis of DPA by inventors Y.S.J and M.Y.

## Figures and Tables

**Table 1:** Effects of reaction time and temperature on di-*ᴅ*-psicose anhydride (DPA) and *ᴅ*-psicose concentrations.

Compound	Temp (°C)	Anhydrous Acidify Reaction (mM) ^1^				
		1 Day	2 Day	4 Day	6 Day	8 Day
Di-*ᴅ*-psicose anhydride	–20	440.9 ± 0.1 ^G^	480.9 ± 0.4 ^FG^	502.9 ± 0.2 ^F^	487.1 ± 0.4 ^FG^	569.2 ± 0.3 ^DE^
	4	521.5 ± 0.1 ^EF^	579.8 ± 0.2 ^D^	668.5 ± 0.3 ^C^	733.1 ± 0.2 ^B^	893.5 ± 0.5 ^A^
	25	719.5 ± 0.3 ^B^	474.0 ± 0.4 ^FG^	N.A	N.A	N.A
	40	N.A ^2^	N.A	N.A	N.A	N.A
*ᴅ*-Psicose	–20	1096.0 ± 0.2 ^AB^	1113.0 ± 0.4 ^A^	972.6 ± 0.3 ^C^	866.9 ± 0.4 ^CD^	929.4 ± 0.7 ^C^
	4	986.3 ± 0.6 ^BC^	881.8 ± 0.3 ^C^	760.3 ± 0.5 ^D^	697.5 ± 0.2 ^E^	738.0 ± 0.6 ^E^
	25	403.0 ± 0.5 ^F^	340.8 ± 0.7 ^F^	N.A	N.A	N.A
	40	N.A	N.A	N.A	N.A	N.A

^1^ Data are expressed as mean ± standard deviation (*n* = 3). Means with different superscripted letters in the same compound significantly differ in terms of Tukey–Kramer honestly significant difference test (*p* < 0.05). ^2^ Not available.

## Data Availability

Not applicable.

## Supplementary Materials

The following supporting information can be downloaded at: https://www.mdpi.com/article/10.3390/ijms232112827/s1.
