# Supplementary material for: Synthesis of a New Glycoconjugate with Di-ᴅ-Psicose Anhydride Structure
Source: Int J Mol Sci. 2022 Oct 24;23(21):12827. doi: 10.3390/ijms232112827 (PMC9655448; doi:10.3390/ijms232112827)
Supplement: Supplementary file 1 [file ijms-23-12827-s001.zip › ijms-1955847-supplementary.pdf]

Supplementary Data

Synthesis of a new glycoconjugate with di-*D*-psicose anhydride structure

Young Sung Jung, Hyoung-Geun Kim, Min-Cheol Lim, Ji-Su Park, Soonok Sa, and Miyoung Yoo*

*Corresponding author: Tel.: +82-63-219-9342; Fax.: +82-63-219-9280; E-mail addresses: myyoo@kfri.re.kr.

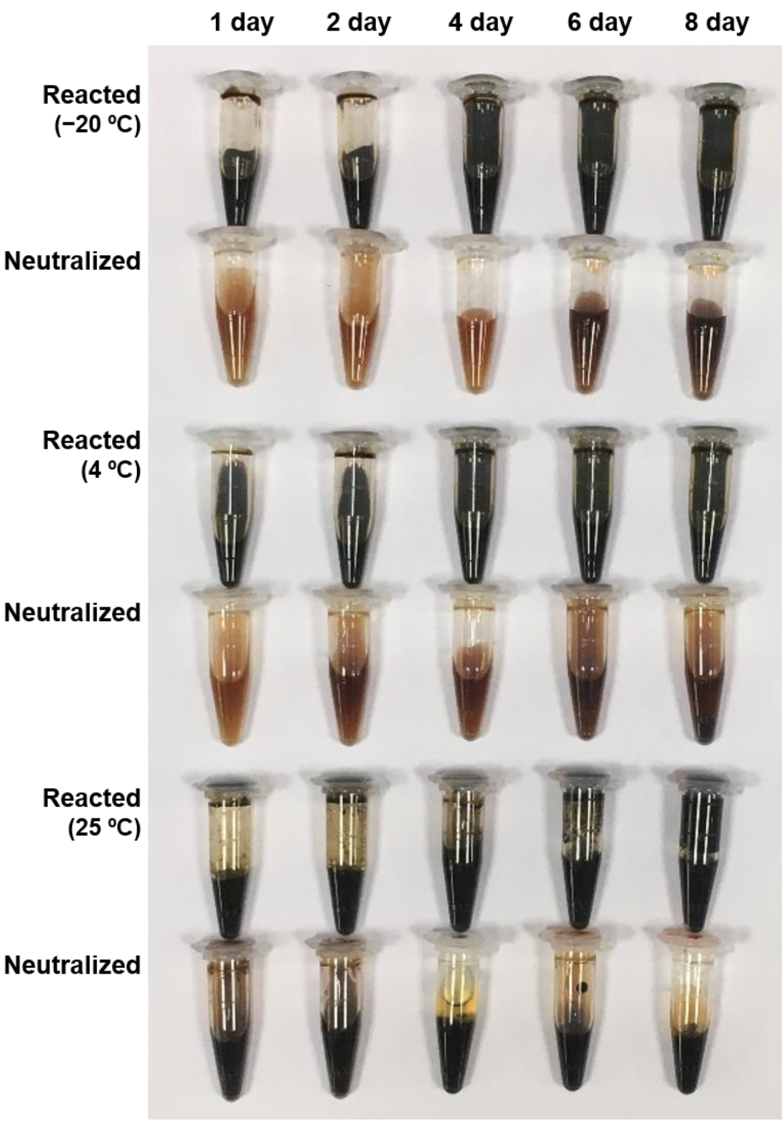

Figure S1. Caramelization of *D*-psicose with time and temperature.

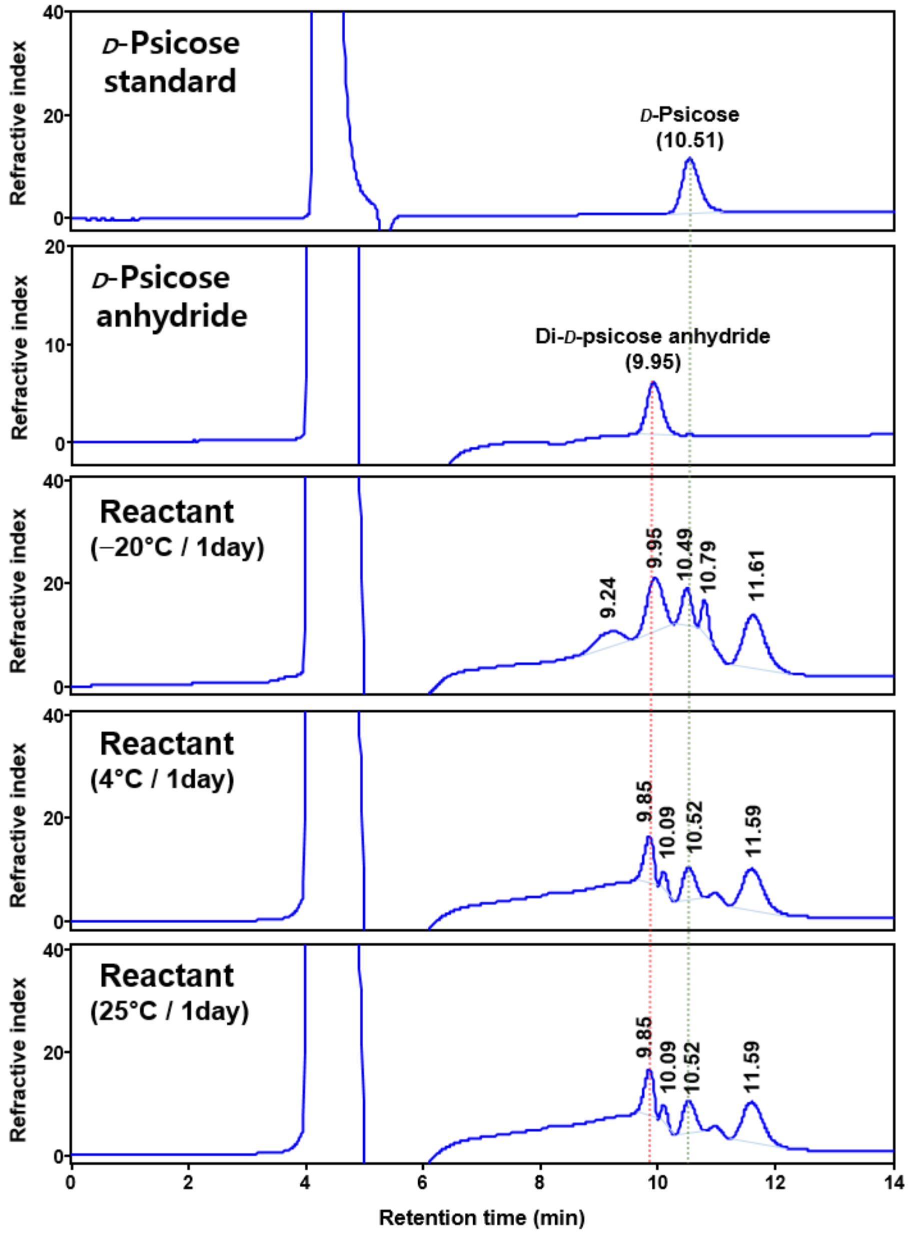

Figure S2. Composition of di-*D*-psicose anhydride (DPA), *D*-psicose, and other peaks with reaction temperature for one day under hydrochloric acid condition.

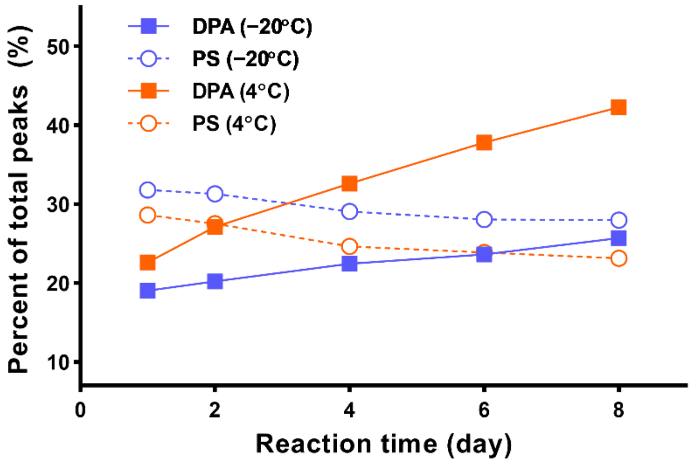

Figure S3. Ratio of di-*D*-psicose anhydride (DPA) production to *D*-psicose residual with reaction time and temperature under hydrochloric acid conditions.

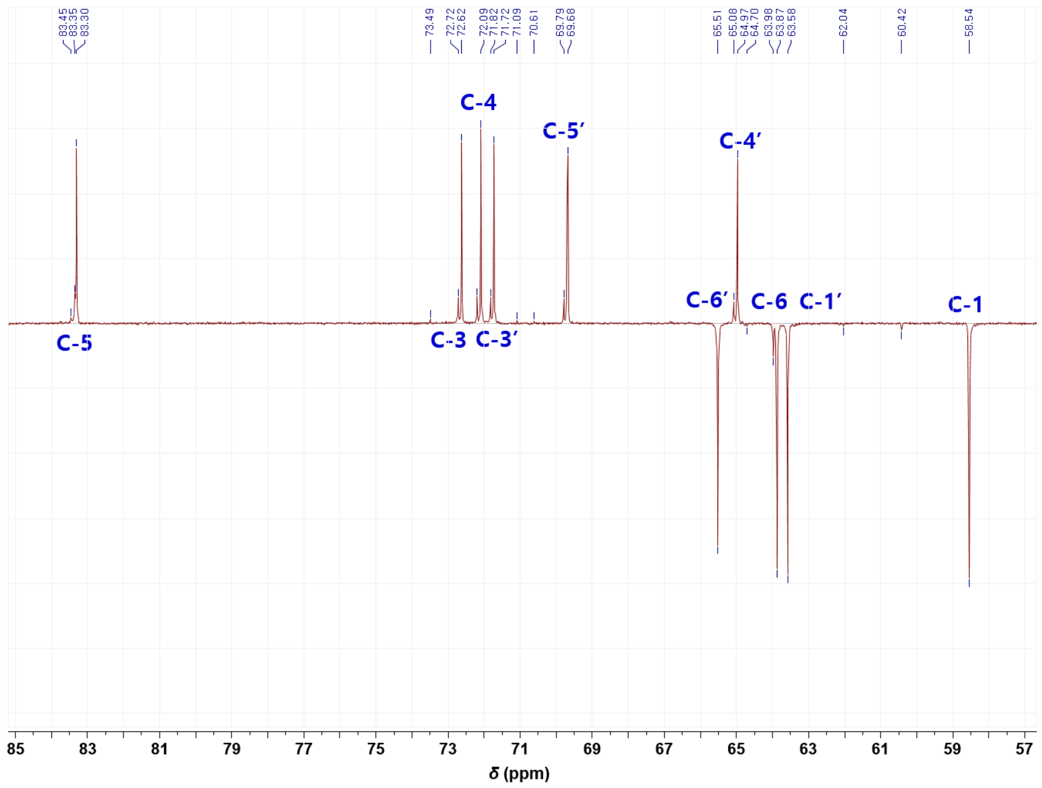

Figure S4. Distortionless enhancement by polarization transfer 135 NMR spectrum of di-*D*-psicose anhydride.

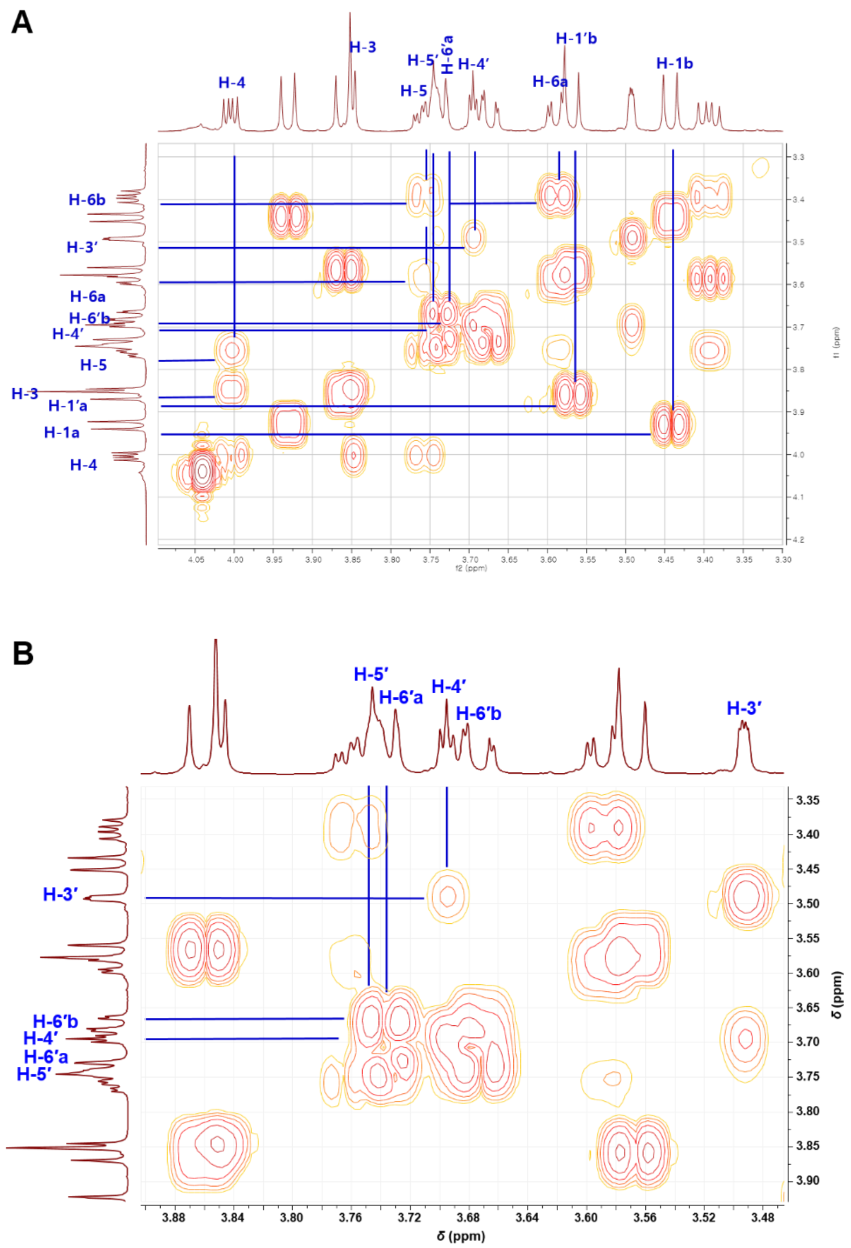

A
H-4
H-3
H-5′
H-6′a
H-4′
H-5
H-6a
H-1′b
H-1b
H-6b
H-3′
H-6a
H-6′b
H-4′
H-5
H-3
H-1′a
H-1a
H-4
f2 (ppm)
f1 (ppm)
B
H-5′
H-6′a
H-4′
H-6′b
H-3′
H-3′
H-6′b
H-4′
H-6′a
H-5′
δ (ppm)
δ (ppm)

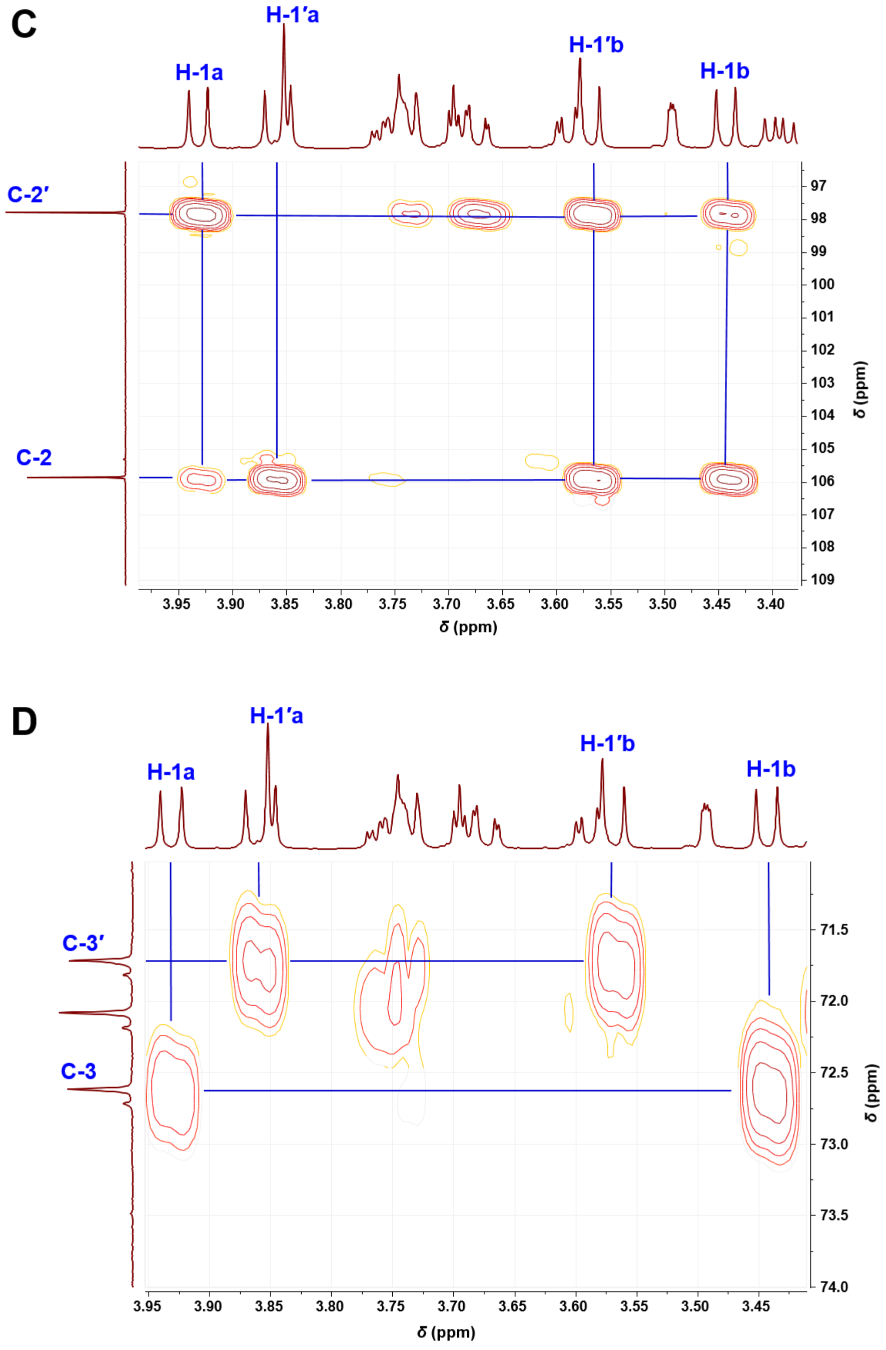

Figure S5. Correlated spectroscopy NMR spectra of di-*D*-psicose anhydride. Full scale (A) and enlarged scale (B). Correlation for C-2 and C-2′ (C) and correlation for C-3 and C-3′ (D).

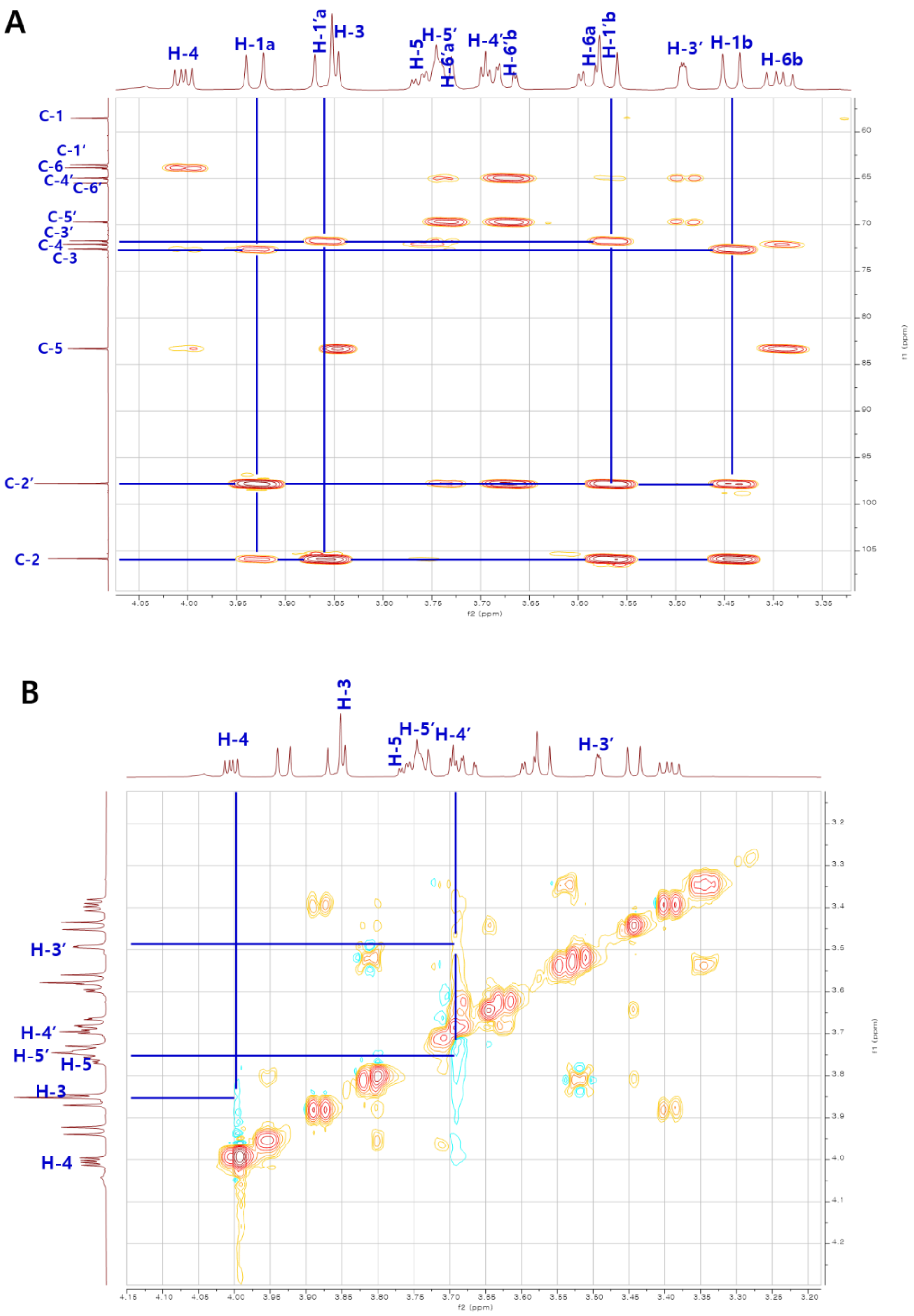

Figure S6. Heteronuclear multiple bond correlation (A) and nuclear overhauser enhancement spectroscopy (B) NMR spectra of di-*D*-psicose anhydride.
